# SIRT1 and Estrogen Signaling Cooperation for Breast Cancer Onset and Progression

**DOI:** 10.3389/fendo.2018.00552

**Published:** 2018-09-27

**Authors:** Sergio Liarte, José Luis Alonso-Romero, Francisco José Nicolás

**Affiliations:** ^1^Laboratorio de Oncología Molecular y TGFβ, Instituto Murciano de Investigaciones Biosanitarias Arrixaca, Murcia, Spain; ^2^Servicio de Oncología, Hospital Clínico Universitario Virgen de la Arrixaca, Murcia, Spain

**Keywords:** SIRT1, sex steroids, estrogen receptor, GPER, breast cancer, TNBC

## Abstract

Breast cancer remains a significant female mortality cause. It constitutes a multifactorial disease for which research on environmental factors offers little help in predicting onset or progression. The pursuit for its foundations by analyzing hormonal changes as a motive for disease development, indicates that increased exposure to estrogens associates with increased risk. A prevalent number of breast cancer cases show dependence on the increased activity of the classic nuclear estrogen receptor (ER) for cell proliferation and survival. SIRT1 is a Type III histone deacetylase which is receiving increasing attention due to its ability to perform activities over relevant non-histone proteins and transcription factors. Interestingly, concomitant SIRT1 overexpression is commonly found in ER-positive breast cancer cases. Both proteins had been shown to directly interact, in a process related to altered intracellular signaling and aberrant transcription, then promoting tumor progression. Moreover, SIRT1 activities had been also linked to estrogenic effects through interaction with the G-protein coupled membrane bound estrogen receptor (GPER). This work aims to summarize present knowledge on the interplay between SIRT1 and ER/GPER for breast cancer onset and progression. Lastly, evidences on the ability of SIRT1 to interact with TGFß signaling, a concurrent pathway significantly involved in breast cancer progression, are reported. The potential of this research field for the development of innovative strategies in the assessment of orphan breast cancer subtypes, such as triple negative breast cancer (TNBC), is discussed.

## Introduction

Breast cancer (BC) is the most frequent tumor in women and a prevailing cause for female cancer mortality (1). It constitutes a multifactorial disease for which epidemiologic studies over environmental determinants offer little help in predicting disease onset or progression, thus, gender, aging, diagnosed first-degree relatives or previous history of BC remain dominant risk factors (2, 3). The pursuit for BC causes over molecular biology techniques led to the establishment of few genetic markers, such as *BRCA1, BRCA2, or DBC-1*, which driver mutation predispose for disease while also explaining cases of familiar clustering; still, the majority of mutations detected account for genes of low penetrance and frequently altered across genomes (4). Whilst, concurrent research efforts tried to find a predictable BC marker or cause based on hormonal changes.Although for most of hormonal hypothesis proposed data is still inconclusive (2, 5), it is accepted that increased exposure to estrogens associates with higher risk, while reducing exposure is believed to result in protection (6). Therefore, factors such as early menarche, nulliparity or late menopause are associated with enhanced BC likelihood.

Observations on BC incidence are thought to be significantly contributed by the activities of classic nuclear hormone receptors for estrogens and progesterone (ER, PR). This is supported by tumor characterization using microarray techniques, which allowed to discriminate BC subtypes on the expression of key molecular markers including classic receptors. Applying such methods, BC is classified in ascending order indicating for aggressiveness: luminal-A; luminal-B (HER2–); luminal-B (HER2+); HER2-enriched; and basal-like. The first three subtypes share positivity for the expression of ER and/or PR along with the presence or absence of the human epidermal growth factor receptor 2 (Erb-B2; HER2), member of the epidermal growth factor receptor (HER/EGFR/ERBB) family. At last, the basal-like, which displays worsened prognosis and develops predominantly in pre-menopausal women, is characterized for the expression of specific basal-epithelium markers such as keratins (7). However, in clinical practice, cost-effective immunohistochemical methods are preferred to determine receptor presence, as such sub-typing, while providing prognostic information, also allow setting therapies to target specific oncogenic markers (Table 1). The anatomopathological absence of ER, PR, and HER2 led to the expression “triple negative” (TNBC), discerning cases devoid for all three markers (15). Worth noting, TNBC is somewhat regarded a surrogate for basal-like, as 70–80% overlap has been described between classifications (8, 16). In any case, while BC triage usually allows for adapted treatments improving prognosis in receptor-positive categories, that does not apply for TNBC cases, for which the lack of targeted approaches frequently restrict options to chemotherapy, with obvious consequences for the prognosis and lethality of the disease (17).

**Table 1:** Breast cancer anatomopathological surrogate definitions based on immunohistochemical subtyping methods.

**Definition marker**	**Luminal A-like**	**Luminal B-like (HER2 negative)**	**Luminal B-like (HER2 positive)**	**HER2 positive (non-luminal)**	**Triple negative (ductal)**
Erb-B2	–^*^	–^*^	+^*^	+^*^	–^*^
ER	+	+	+	–	–
PR	+	–/↓	+	–	–
Other relevant	Ki-67↓	Ki-67↑	Ki-67		Cytokeratins^**^
Prevalence	30~70%	10~20%		10~20%	15~25%
Main treatment strategy	Endocrine therapy	Endocrine therapyCytotoxic		anti-HER2 Cytotoxic	Cytotoxic
Recurrence Risk	↓	↑		↓	↑↑
SIRT1	~74%			~55%	~42%

Concurrent marker detection is achieved for establishing subtypes. Not mentioned minor special histological types may respond to targeted therapies. Erb-B2, human epidermal growth factor receptor 2 (HER2);

* overexpressed or amplified; ER, Estrogen Receptor; PR, Progesterone Receptor; Ki-67, proliferation marker; Cytokeratins, basal-like marker (8–14).

** *highly overexpressed; ^↓^low expression/risk; ^↑^high expression/risk; ^↑↑^very high expression/risk*.

Interest on histone deacetylases (HDACs) is expanding as accumulated findings highlight their impact on regular physiology and pathological condition, staying SIRT1 the most studied (18). Sirtuins comprise a family of proteins (SIRT1–7) described as type III HDACs relying on NAD+ availability to perform a gatekeeping role in the configuration of the cell transcriptome, function that appears highly dependent on the cellular context and has been involved in a variety of biological processes, from modulating energy metabolism to development and cellular senescence (19–21). Intriguingly, Sirtuins in general and SIRT1 in particular display a paradoxical role in cancer, with histological studies showing increased or decreased expression patterns upon cancer origin and/or stage (22–24). In that sense, numerous contributions support the notion that SIRT1 activities influence hormone receptors (HR) actions, expressly those mediating long-term estrogenic effects in the mammary gland, namely the classic ERs, for which a relevant degree of interdependence between these factors has been described with apparent importance for BC onset and development. Moreover, interaction between the anew membrane-bound G-protein coupled estrogen receptor (GPER), which ubiquitously mediates short-term estrogenic effects, and SIRT1 has been also proposed, an interplay which could help fostering BC survivability and progression. Altogether, the conjoint actions of SIRT1 and HRs pose deep implications for BC onset and progression, which turn significantly relevant for the case of drug-resistant cases and conceivably HER2-enriched and TNBC. From that scope, this review synthetizes current knowledge on this emerging field.

## Known implications of SIRT1 for cancer onset and progression

Initially described to deacetylate histones H1, H2, and H4 (18), SIRT1 is supposed to contribute to chromatin remodeling beneficial for tumor progression. However, despite obvious epigenetic capabilities, HDACs and SIRT1 current relevance for cancer strive on their now known ability to act on different substrates. SIRT1 is regarded an established modulator of significant non-histone nuclear proteins, such as p53, E2F1, or NF-kB (25, 26). SIRT1 overexpression has been related to tumor cell survival through the deacetylation and subsequent degradation of the p53 tumor suppressor (27, 28). Additionally, SIRT1 is known to deacetylate the FOXO family of transcription factors, resulting in repression of pro-apoptotic elements (29–31). In this line, using both hormone-responsive and TNBC models, MCF-7 and MDA-MB-231 cell lines respectively, SIRT1 has been found to localize to the promoters of silenced tumor suppressor genes, state reverted upon SIRT1 activity inhibition (32). Additionally, there is open debate on the ability of SIRT1 to act over cytosolic targets, with proposed tumorigenic implications related to PI3K/IGF-1R signaling (33, 34).

Moreover, SIRT1 shows a convoluted role in the regulation of the epithelial-mesenchymal transition (EMT) process, with apparent relevance in the case of reproductive tumors such as prostate and BC (35, 36). Notably, SIRT1 has been found to upregulate the expression of matrix-metalloproteinases in BC cells, condition known to promote invasiveness (37, 38). Moreover, SIRT1 activities seem to coordinate cancer stem cell-EMT changeover through deacetylation of a complex circuitry of transcription factors (39). To this regard, SIRT1 has been recently found to interact with Smad proteins, TGFß signaling canonical transducers (40). Interestingly, TGFß deregulation is regarded a cornerstone for EMT and tumor dispersion, among others markers, affecting the expression of matrix-metalloproteinases (4). While having a demonstrated role on the degradation of the inhibitory-Smad Smad7, SIRT1 has been also shown to relate with receptor-regulated Smads (Smad2 and Smad3), possibly coactivator-Smad Smad4 as well, in a process linked to altered transcriptional output (36, 40–42). Consequently, such interplay prompts for wide implications on cell transformation and tumor progression/dispersion.

## Estrogen signaling and breast cancer: the ER pathway

Among steroids, estrogens comprise a set of hormones involved in the development and maintenance of the female reproductive system, the main representative of which is 17ß-estradiol (E2), constituting a major hormonal input along the monthly cycle. For its synthesis, androgens are converted into estrogens on the action of CYP19A1, known as Aromatase, an enzyme being mainly expressed in the ovaries but found in other tissues including the mammary gland (43). ERα and ERß correspond with the classic HRs responsible for E2 long-term effects (44). Both receptors, along with PR, integrate into the nuclear receptor family, which include sex steroids receptors as well as receptors for corticosteroids (45). Its members mostly localize to the nucleoplasm, but a minority of isoforms which help fine-modulating the overall response may also appear at alternate locations, involving a cytoplasmic-nuclear shuttling mechanism (46, 47). Although ERα and ERß share significant sequence homology, both receptors display unique expression patterns depending on either tissue or organ. ERα signaling comes crucial for the regulation of mammary gland development and function (44), also contributing to cancer onset and progression. Elevated ERα levels expressed in benign breast epithelium correlate with enhanced BC risk, whereas estrogen-dependent cancers require of E2 for cell survival and growth (44). Worth noting, PR positivity can be usually regarded a surrogate of ER positivity, as PR expression requires proper ER functioning to occur (48).

The principal ER activation mechanism requires E2 binding, which allows conformational changes promoting receptor dimerization and nuclear translocation to interact with estrogen response elements (ERE) present in the DNA to regulate transcription (44). Additionally, alternate mechanisms for ER activation and modulation had been described through cross-linking with signaling pathways like EGFR/PI3K/ERK, based on changes of ER phosphorylation status (49, 50). Upon landing on EREs, E2-ER complexes further recruit several co-activator proteins such as p300, PPARγ, and PGCα, which leads to histone acetylation and chromatin remodeling necessary for the regulation of transcription (51). Moreover, active ERα also recruit members of the FOXO family into transcriptomic complexes, interplay that has been characterized to greatly influence transcriptomic outcomes, with implications for mammary morphogenesis, BC onset and progression to drug resistant states (52–56).

At the clinic, hormone-responsive BC is usually managed in a straightforward manner due to the multiplicity of pharmacological approaches based in the use of Aromatase inhibitors along with selective ER modulators and downregulators, like Tamoxifen or Fulvestrant, which diminish ER-mediated E2-responses at the breast (57). Consequently, these cases are mostly associated with favorable prognosis. Still, hormone-responsive tumors frequently transform over time into an estrogen-independence status, gaining the ability to proliferate in the absence of hormonal input. This conversion usually becomes a critical step for BC clinical progression, as it is related to increased aggressiveness. In that sense, ER-negative cases at diagnosis are believed to have lost ERα expression over time due to gene silencing, thus reducing therapeutic options targeting HRs and thus associating with less favorable prognosis (58).

## Estrogen signaling and breast cancer: the GPER pathway

Despite the fact that classical HRs may be missing for some BC cases, however, it is now acknowledged that estrogen stimulation would still play a powerful role for the evolution of the disease through the actions of membrane-coupled ERs. Turning into the XXI century, the existence of a membrane-coupled ER was revealed, that would be responsible for most of E2 rapid physiological responses which at the time lacked of proper molecular explanation. This marker corresponded with a G-protein coupled receptor which could be located both at the endoplasmic reticulum and the plasmatic membranes (59, 60). Initial reports indicated that through its binding to E2, GPER actions resulted in cAMP and inositol triphosphate production, triggering intracellular calcium mobilization. However, it was rapidly established that its actions also resulted in the transactivation of diverse intracellular signaling cascades including MAP-Kinases, PI3K, or eNOS (61–63). In this line, further research also found GPER able to attune E2-mediated transcription regulation. This was established on co-expression experiments showing the existence of a functional cooperation between canonical-ERα-mediated and short-time-GPER-mediated signaling through a mechanism relying on the activation MAP-Kinases, capable of promoting post-translational modifications such as altering ERα phosphorylation status (64). Moreover, additional studies established GPER to be ubiquitously expressed through both reproductive and non-reproductive tissues (65), thus posing new challenges for the understanding of estrogen physiology. In that sense, it was not long before GPER was proposed to contribute for cancer development (61). Still, GPER's specific role for both neoplastic transformation and cancer progression remains unclear, as its actions seem to be highly depend on tissue origin (66). Yet, although anatomopathological studies on GPER expression are not included in routine clinical practices, in the case of estrogen-dependent tumors and especially BC, several works show that its altered expression can be associated with cancer progression, supporting a potential prognostic value (67–69).

At the molecular level, diverse studies advocate for GPER's ability to affect cancer cell survivability and proliferation, by influencing a myriad of signaling pathways responsible for cell cycle regulation or apoptosis control. They also play a role in the regulation of angiogenesis required for tumor nourishment and development [reviewed at (66)]. Moreover, GPER signaling has been proposed to distinctly affect cell migration and cancer invasiveness. Calpains are non-lysosomal proteases that are implicated in the regulation of cell adherence to the extracellular matrix and motility (70). Interestingly, it was reported that incubation of ER-negative BC cells with GPER agonists G-1 promoted Calpain-1 activity and altered adhesion to matrigel (71). Also in this line, GPER has been described to adjust EGFR/PI3K/ERK intracellular signaling, favoring expression changes of migration markers such as SNAIL or ß1-integrin, as well as altering plasticity of cellular adhesions by the activation of the focal adhesion kinase (FAK) (72). Conjointly, these abilities of GPER to promote viability and motility provide a molecular framework for estrogen stimulation of cancer cells despite ER-negativity. Notably, epidemiologic studies would deem clinical functionality to these capacities, as prevalence for GPER overexpression is found in HER2-enriched, basal-like and TNBC cases, also associated with both distant metastasis and recurrence (67, 73).

## Estrogen signaling and breast cancer: noted SIRT1 influence

Numerous contributions support the notion that SIRT1 activities decisively impact HRs actions in relation to disease. With regard to classic ERs, in cellular models, E2 stimulation has been found to promote SIRT1 expression under direct influence of ERα transcriptomic complexes, observation matched by the prevalent detection of elevated levels for SIRT1 manifested for most of ERα-positive BC samples (74, 75). Interestingly, the ability of SIRT1 to interact with p300, PPARγ, and PGCα, ERα transcriptomic co-activators, has been proved to affect chromatin remodeling, disturbing developmental processes (76–78). Following studies found SIRT1 inhibition resulted in suppressed ERα expression, interfering with E2-dependent cell growth in healthy as well as malignant mammary epithelial cells (79, 80). In this line, inhibition of SIRT1 has been found to lessen ERα mediated repression of NRF2-dependent detoxifying enzymes in MCF-7 cells (81).

Simultaneously, significant efforts had been put to apprehend the effects of a less subtle SIRT1-ER interaction mechanism. Interestingly, ERα has been found to relate to and be a target for SIRT1. Upon E2-ERα activation, p300 stabilizes receptor complexes through acetylation in a process that can be reversed by SIRT1 (82). These findings initially suggested a SIRT1 inhibitory role over estrogen signaling. Still, changes in ERα acetylation status are considered to have limited effects (83). On the other hand, as previously mentioned, active ERα recruits FOXO family members into transcriptomic complexes, factors which also fall under SIRT1 spectrum of actions. Within the BC context, a recent report showed FOXN3 to be able to recruit SIRT1 into ERE-dependent transcriptomic complexes, promoting reduced transcriptomic output (84). Interestingly, alterations of FOXM1 and FOXO3a levels had been previously linked to SIRT1 aberrant activity, especially in TNBC (85).

Regarding the interaction between SIRT1 and GPER, knowledge is scarce. Nonetheless, using ER-negative HER2-enriched BC cells, it was recently described that E2 actions via GPER can result in SIRT1 overexpression through activation of the EGFR/ERK/c-fos/AP-1 transduction pathway (86). Interestingly, such induction contributed specifically to enhanced cancer cell survival and proliferation, as it was reversed by the use of SIRT1 inhibitors or GPER silencing as well. Notably, these effects were also observed in cancer associated fibroblast obtained from BC patients (86), posing challenging questions for this interplay in BC progression.

## Present and future clinical value of the estrogen signaling/SIRT1 interplay

Finding ways to better screen and characterize BC, especially drug-resistant cases and TNBC, constitutes a standing challenge of cancer research. Efforts to establish a SIRT1 prognostic value for BC are increasing, as its overexpression can be commonly detected (9). Recent meta-analysis gathering non-connected data sources, while finding association with higher tumor stage, failed to detect correlation between SIRT1 expression levels and BC overall survival; however, these works addressed statistics letting out BC subtyping (87). Notwithstanding, a single retrospective study incorporating 822 BC patients found SIRT1 expression to correlate with tumor aggressiveness and reduced disease-free-survival (DFS) (88). Interestingly, when BC subtyping is considered, SIRT1 overexpression associates with ER-positivity and likely shortened DFS (9). Moreover, while not associated with HER2-enriched or TNBC status, concomitant SIRT1 overexpression successfully dictates for lymph-node metastasis likelihood (9), observation which has been linked to the ability of SIRT1 to promote an altered expression of key EMT markers such as E-Cadherin, Vimentin, and SNAIL-1 (89). Hence, although data so far may be considered insufficient or poorly curated, the accumulated evidence allows to consider SIRT1 histological detection as a valuable marker for assessing BC status, also providing hints on metastasis and relapse odds.

Per BC tumor progression, it is speculated whether SIRT1 has a role in determining tumor conversion to a drug-refractory phenotype. It has been reported that SIRT1 activity helps increasing expression of drug-resistance genes (90), in a process that involves FOXO1 deacetylation and reverts upon SIRT1 suppression (91, 92). Noteworthy, SIRT1 activity is mainly regulated by upholding absolute protein levels with little variation overtime (35), although modulation of its actions depending on MAPK-mediated phosphorylation has been also described (93). To this extent, two considerations should be made. Firstly, it ought to be reminded that estrogen signaling via either HR can augment SIRT1 levels (74, 86). Interestingly, elevated SIRT1 levels has been reported to promote increased Aromatase activity both in ER-positive and TNBC cell lines (94), thereby allowing for local E2 production. Secondly, it should be noted that MAP-kinases signaling can be activated by overexpressed HER2 subunits dimerizing with EGFR and triggering the PI3K/ERK pathway (95). Consequently, a SIRT1 potential role assisting evolution to ER-independence turns reasonable, as deleterious abilities, including aberrant modulation of p53 and FOXO or expression of drug-resistance genes, could be maintained via SIRT1 overexpression depending on GPER signaling, perhaps also through MAP-kinases dependent activation. In that sense, recent reports showing linkage between SIRT1 overexpression and enhanced SRC and AKT activities in different TNBC cell lines would support the latter notion (96, 97). Worth considering as well, classic ERs and GPER display dissimilar behavior upon exposure to pharmaceutical modulators like Tamoxifen and Fulvestrant, which in the case of GPER appear to have an agonistic function (66). Moreover, in cellular models, GPER activation has been described to negatively affect ERα protein levels (71), perhaps fostering the transition. Hence, considering that solid tumors are composed by a clones plethora subject to selective pressure, it is tempting to propose the monitoring of SIRT1 expression/activity as source of information on the efficiency of treatments related to tumor evolution at the cellular scale. To this regard, procedures based on liquid-biopsy techniques would offer an appropriate framework for such approach (98).

On the aspect of treatments, many efforts had tried to assess the potential of suppressing SIRT1 activities. However, after several years, effects described on the many inhibitors discovered and interference studies do not agree with each other. This is regarded as the result of dissimilar mechanistic involved in knocking-out or knocking-down the enzymatic activity and also on the specificity of inhibitors used (99). Yet, modulators of SIRT1 activity may still retain potential as co-adjuvant treatments, due to sensitizing capabilities useful at specific BC environments (100). In this sense, sirtuins in general, and SIRT1 in particular, have been shown to interact with numerous signaling pathways which decisively affect different aspects of the cell physiology. That is the case with the recently described interaction of SIRT1 with TGFß canonical-signaling-transducers, the Smad proteins. SIRT1 has been found incorporated into Smad-mediated transcriptomic complexes, its activity linked to reduced Smad acetylation and decreased nuclear half-life (36, 40–42). Lack of proper TGFß response is considered a major mechanism for EMT through regulating the expression of key cell adherence and migration markers (4). Interestingly, this TGFß dependent regulation involves the participation of FOXO factors (101, 102). Hence, the overexpression of SIRT1 in BC cells has the potential to facilitate aberrant regulation through these system, thus contributing for the EMT process and cancer progression. Moreover, as signaling via GPER and HER2 are known to trigger MAP-Kinases activation, and activated MAP-Kinases had been related to SIRT1 augmented activity, this interplay provides an additional framework for the promotion of both tumor ER-independence and EMT progression, potentially offering opportunity to develop tailored strategies, which would come particularly useful for the case of TNBC. Perhaps, next-generation inhibitors, with better specificity and increased potency, may provide advancements in this field.

Finally, a brief but necessary mention should be made on non-coding RNAs. Increasing efforts attempt to discriminate the relevance of both long-non-coding and micro-RNAs in BC pathogenesis and progression, due to their dual role as an additional prognostic information source and potential therapeutic targets (103, 104). Not surprisingly, distinct signatures could be found in BC depending ER status (105). In that sense, exposure to either estrogens or selective ER modulators had been shown extensively affect through classic ERs the microRNA profile of different mammary cell lines (106). Interestingly, within the BC context, SIRT1 activity appears to be highly conditioned by the non-coding-RNA environment, with several long- and microRNA species affected by endocrine signaling being able to promote or downregulate its expression (107, 108). Further research in this area may again probe helpful for the development of strategies targeting intrinsic BC subtypes.

## Concluding remarks

**Figure 1:**
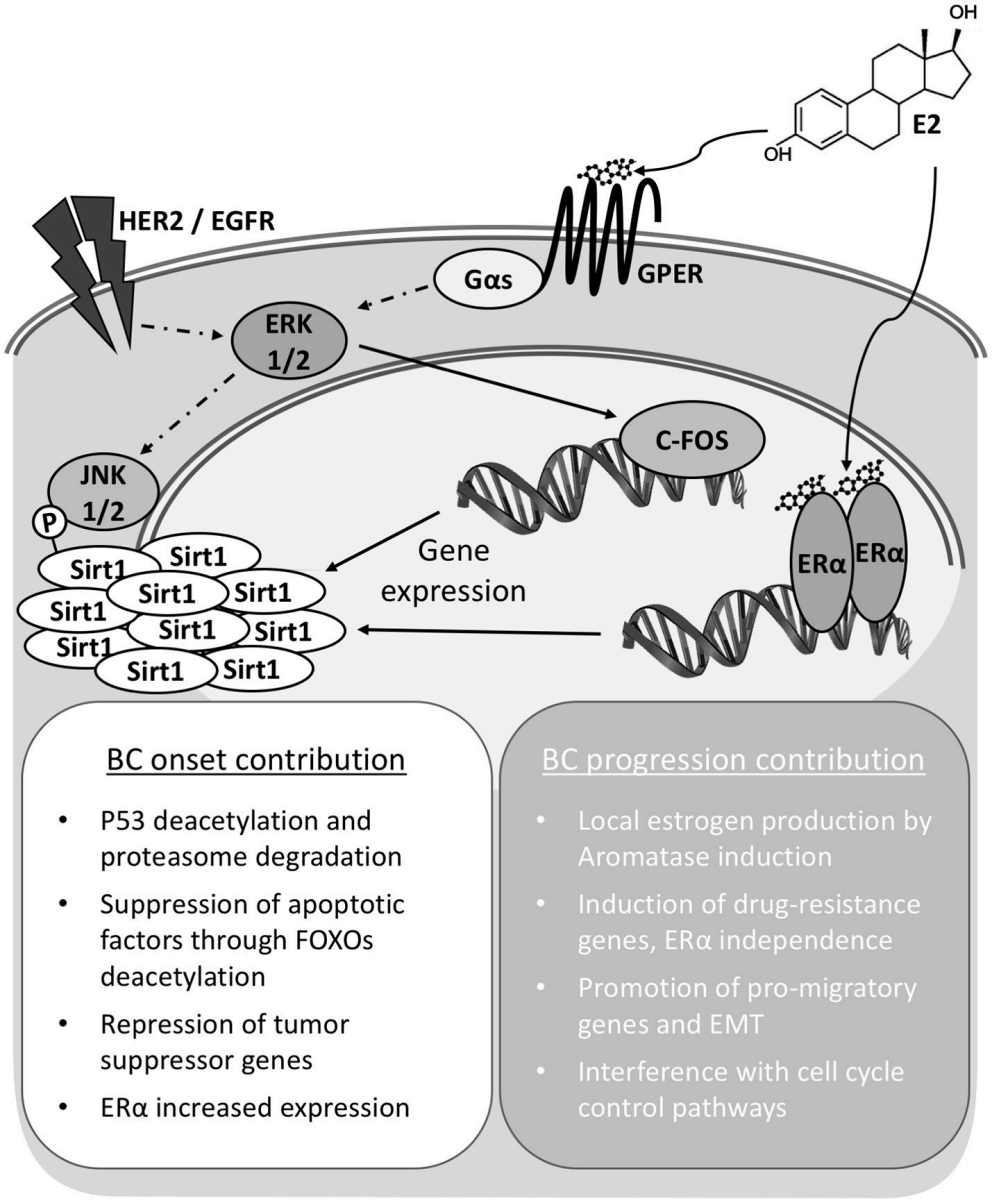
Sirt1 overexpression contributes to Breast Cancer onset and progression. In breast cancer cells Sirt1 overexpression can be achieved independently through either nuclear (ERα) or membrane bound G-protein coupled estrogen receptor (GPER) signaling. Total activity is also modulated by phosphorylation status. Increased Sirt1 activities and interaction with diverse factors result in pleiotropic effects supporting cell survival and transformation for cancer onset. Continued activity contributes to cell de-differentiation and epithelium to mesenchyme transition (EMT).

The accumulated evidence prompts to consider SIRT1 as an integrated player into the transduction network activated by estrogens, both through ERs and GPER, in the mammary tissue (Figure 1). This tight cooperation conceptualizes and supports a SIRT1 promoting role in mammary tumorigenesis, with meaning for both disease onset and progression. These implications are to be considered both in the case of signaling triggered upon endogenous estrogens exposure, also in the case of exposure to environmental pollutants like xenoestrogens, phytoestrogens, and other synthetic compounds; let aside for the response to BC treatments based on estrogen modulators. Consequently, SIRT1 detection has potential to become a powerful prognostic indicator for tumor evolution and response to chemotherapeutics. Moreover, a better understanding at the molecular level of its cooperation and impact in the signaling through connected pathways may provide opportunity for the development of innovative therapy approaches in the assessment of BC and particularly TNBC cases.

## Author contributions

SL conceptualized the work, performed searches in literature databases and prepared, reviewed and edited the original draft of the manuscript. JA-R contributed with formal analysis and the incorporation of clinical aspects, also reviewed the original draft. FN supervised the project, was responsible for funding acquisition and reviewed and edited the original draft. All authors read and approved the final manuscript.

### Conflict of interest statement

The authors declare that the research was conducted in the absence of any commercial or financial relationships that could be construed as a potential conflict of interest.
